# High-throughput DFT calculations of formation energy, stability and oxygen vacancy formation energy of ABO_3_ perovskites

**DOI:** 10.1038/sdata.2017.153

**Published:** 2017-10-17

**Authors:** Antoine A. Emery, Chris Wolverton

**Affiliations:** 1 Department of Materials Science and Engineering, Northwestern University, Evanston, Illinois 60208, USA

**Keywords:** Atomistic models, Electronic structure, Density functional theory

## Abstract

ABO_3_ perovskites are oxide materials that are used for a variety of applications such as solid oxide fuel cells, piezo-, ferro-electricity and water splitting. Due to their remarkable stability with respect to cation substitution, new compounds for such applications potentially await discovery. In this work, we present an exhaustive dataset of formation energies of 5,329 cubic and distorted perovskites that were calculated using first-principles density functional theory. In addition to formation energies, several additional properties such as oxidation states, band gap, oxygen vacancy formation energy, and thermodynamic stability with respect to all phases in the Open Quantum Materials Database are also made publicly available. This large dataset for this ubiquitous crystal structure type contains 395 perovskites that are predicted to be thermodynamically stable, of which many have not yet been experimentally reported, and therefore represent theoretical predictions. The dataset thus opens avenues for future use, including materials discovery in many research-active areas.

## Background & Summary

Due to their large tolerance to oxygen vacancy, ABO_3_ perovskites are widely used for a variety of applications such as solid oxide fuel cells, piezo-, ferro-electricity and thermochemical water splitting^1–3^. Furthermore, their remarkable structural stability with respect to their constituent elements suggests that potential new compounds remain to be discovered. As the number of possible ABO_3_ compounds is large, we use high-throughput density functional theory (HT-DFT) to compute the thermodynamical stability of 5,329 compositions in an exhaustive manner. In addition to the compounds stability, we also calculate the oxygen vacancy formation energies as it is a relevant quantity for many applications involving reduction of these compounds^1–3^. Due to their versatility, perovskites have been studied in several high-throughput works^4–7^. However, to our knowledge, those datasets are not publicly and/or easily available.

**Figure 1:**
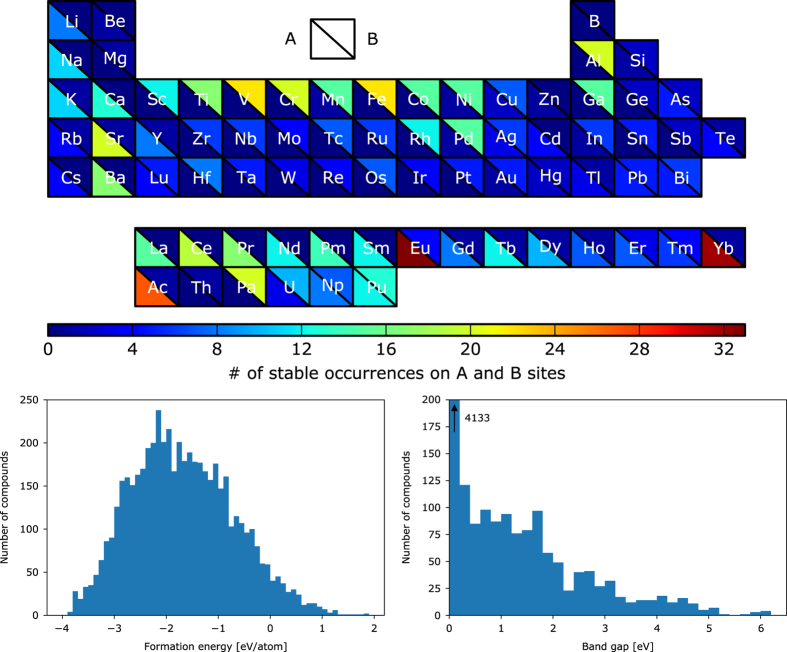
List of elements considered for the A and B sites. Elements are color-coded as a function of the number of stable perovskites with the respective elements on the A and B sites. (Bottom) Histogram representation of formation energies and band gap of compounds calculated in this work.

**Figure 2:**
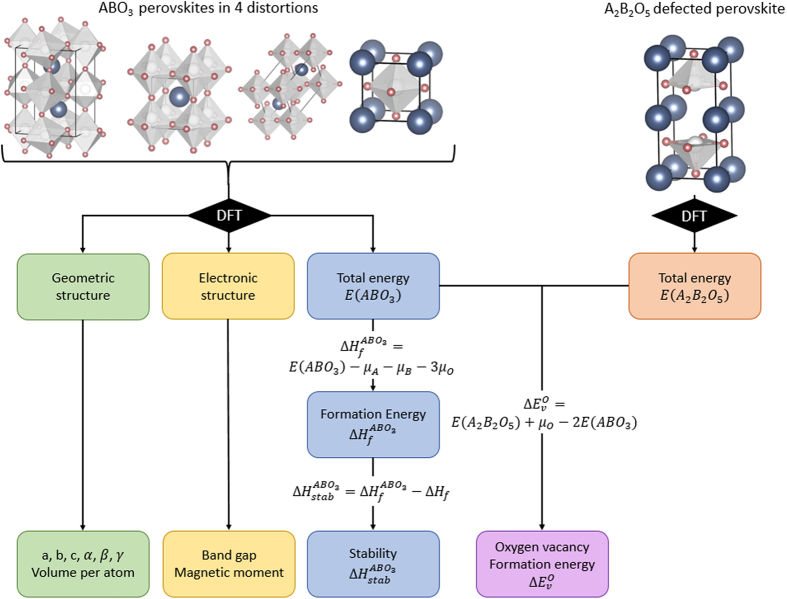
Workflow to calculate all the properties in the current dataset. (top left) We start with all the cubic structures and compute all their total energies using density functional theory (DFT). If the stability of the cubic perovskite (defined in Equation 2) is less than 0.5 eV per atom (i.e., the cubic phase is within 0.5 eV per atom of the ground state convex hull), we also compute 3 additional distortions (orthorhombic, tetragonal, rhombohedral). The geometric properties (lattice parameters, angles, and volume per atom) and electronic properties (band gap and magnetic moment) are readily available from the calculations. Formation energies are calculated using elemental chemical potentials and thermodynamic stability is calculated with respect to all the other A-B-O phases present in the OQMD. (top right) Defected perovskites, a 2×1×1 supercell with a missing oxygen atom, are calculated using DFT and their total energies, in conjunction with those of pristine cubic cells, are used to compute the oxygen vacancy formation energies.

All the 5,329 compounds are created by substituting 73 metals and semi-metals of the periodic table of the elements (see Fig. 1) on the A and B sites (73^2^=5,329) of the ABO_3_ perovskite crystal structure. The ideal ABO_3_ cubic perovskite crystal structure is composed of a B cation that is octahedrally 6-fold coordinated with oxygen atoms and an A cation that is 12-fold coordinated by oxygen atoms. Aside from the ideal cubic structure, many perovskites undergo a local distortion from this cubic structure, and these distorted perovskites can have a variety of symmetries, including the rhombohedral, tetragonal and orthorhombic distortions^3^ (see Fig. 2). In this work, all 5,329 compositions are calculated in the ideal cubic structure and a subset of those (see structural distortions paragraph in the Methods section) are calculated in the 3 aforementioned distortions.

The T=0 K, P=0 bar ground state stability of all ABO_3_ compounds was assessed with respect to all possible linear combinations of phases present in the A-B-O ternary phase diagram using a convex hull construction. All the phases that are used for the stability calculation are from the Open Quantum Materials Database (OQMD)^8^ and (as of July 2017) include ~40,000 phases from the Inorganic Crystal Structure Database (ICSD)^9,10^ and ~430,000 hypothetical compounds based on decoration of common structural prototypes. The oxygen vacancy formation energy was calculated by using an A_2_B_2_O_5_ structure, which corresponds to 2 perovskite unit cells with an oxygen atom removed. Additionally, other properties readily available from DFT calculations are reported, including the relaxed structure, band gap, and total magnetic moment. Figure 2 shows the workflow used to obtain all the quantities.

The present dataset was used in a study aiming at identifying suitable perovskites for thermochemical water-splitting applications using both the stability and oxygen vacancy formation energy as screening parameters^11^. This data is valuable more generally in guiding experimental synthesis of predicted new compounds, further screening for a large variety of applications (other than water splitting) or to train machine learning (ML) models. While machine learning on materials dataset is an area of active research^12–14^, the datasets used by various research groups are often vastly different from one another with no way to compare various ML models. Having a large, consistent materials dataset that can be used by a variety of research groups to train machine learning models will allow a more transparent comparison of various methods being used in the field.

## Methods

### Density functional theory calculations

All compounds were calculated by density functional theory using the Vienna Ab initio simulation package (VASP)^15,16^. The projector-augmented wave method (PAW)^17^ and GGA-PBE^18^, as an approximation to the exchange-correlation functional, were used throughout. 3d transition metals and most actinides were calculated using the DFT+U^19,20^ formalism (see Table 1 for U-values, U is applied on d-electrons for transition metals and f-electrons for actinides) and were spin-polarized with a ferromagnetic alignment of spin. Calculations were performed within the framework of the Open Quantum Materials Database^8^ which contains 470,000 different phases from experimental databases such as the Inorganic Crystal Structure Database (ICSD)^9,10^ and hypothetical compounds based on common structural prototypes.

### Calculation of formation energy

The formation energy of a compound, $H_{f}^{ABO_{3}}$, is calculated according to Equation 1:

(1)HfABO3=E(ABO3)−µA−µB−3µO

where *E*(*ABO*_3_) is the total energy of the perovskites and *μ*_*A*_, *μ*_*B*_ and *μ*_*O*_ are the chemical potentials of A, B and oxygen, respectively. For most elements, chemical potentials are equal to the DFT total energies of their ground states. For some elements where the T=0 K DFT ground state is not an adequate reference state, we apply corrections to the chemical potentials: diatomic gases (O), room temperature liquid (Hg), several elements with structural phase transformations between 0 and 298 K (Na, Ti, Sn) and elements with DFT+U correction (see Table 1). For these elements, chemical potentials are fitted to experimental formation energies. Details of the experimental formation energies used and the resulting corrections are given in ref. 8.

### Calculation of phase stability

Thermodynamic stability was assessed using an energy convex hull construction^21,22^. By definition, the convex hull consists of phases that have an energy lower than any other phase or linear combination of phases at the respective compositions. The stability, or convex hull distance is defined as

(2)HstabABO3=HfABO3−Hf

swhere $H_{f}^{ABO_{3}}$ is the formation energy of the perovskite, defined in Equation 1 and *H*_*f*_ is the convex hull energy at the ABO_3_ composition. For a given phase, P, the convex hull distance is positive if P is unstable and represents the energy difference between P and the convex hull at that composition. The convex hull distance is negative if P is stable and represents the energy difference between P and the (hypothetical) convex hull calculated without compound P. However, in this work, we wish to slightly loosen this restriction to account for nearly-stable compounds and possible uncertainties/errors associated with DFT. Hence, in this paper, we label compounds with a stability below 0.025 eV per atom (approximately kT at room temperature) as stable.

### Structural distortions

After calculating all cubic perovskites, we investigated the effect of distortions on the stability of perovskites. To do so, we randomly selected one-third of the compositions (1,776) and computed their stability in the rhombohedral, tetragonal and orthorhombic distortion. We saw that distortions generally lower the energy of the ideal cubic structure but found no case where the distorted compound was lower in energy than the cubic phase by more than 0.5 eV per atom. Thus, we only calculated the distortion of compositions having a cubic stability lower than 0.5 eV per atom. This resulted in 2,162 (1,776+386) compositions where the four distortions (cubic rhombohedral, tetragonal and orthorhombic) where calculated.

### Calculation of oxygen vacancy formation energy

Oxygen vacancy formation energies are calculated using a cubic undistorted A_2_B_2_O_5_ 9-atom supercell and is given by

(3)EvO=E(A2B2O5)+µO−2E(ABO3)

Where *E*(*A*_2_*B*_2_*O*_5_), *E*(*ABO*_3_) are the DFT total energies of the defect and pristine cell, respectively and *μ*_*O*_ is the chemical potential of oxygen. The choice of 9-atom supercell enables the high-throughput calculation of oxygen vacancy formation energy of all compounds.

### Oxidation states and ionic size

Oxidation states for elements were computed using a bond valence method^23^. If a compound contains at least one element that does not have a BV parameter, the valence of that compound cannot be determined. With the oxidation numbers and coordination numbers (12, 6 and 2 for the A, B and O atoms, respectively) at hand, Shannon radii^24,25^ were used as a measure of the ionic size of every ion.

### Experimental data on stable perovskites

To compare our stability predictions with experimental data, we collected data from the literature on experimentally known perovskites. Four review papers^26–29^ were aggregated and curated to identify 223 ABO_3_ perovskite compositions that have been experimentally synthesized. The curation was made by careful examination of source papers to ensure that the compounds were really synthesized and accurately characterized as a perovskite. In addition, compounds that were synthesized at high pressure (>1 GPa) were removed. We note that, for most of those compounds, the most stable distortion or oxidation states of the elements are not reported in the aggregated review papers.

### Code availability

The automation of the high-throughput calculations and thermodynamic analysis were done using the qmpy python package available at (https://github.com/wolverton-research-group/qmpy) and is released under the MIT license. qmpy was also used to manage the high-throughput workflow and visualize the output from calculations. VASP, the DFT code used to generate the data in this work, is a proprietary software available at http://www.vasp.at.

## Data Records

The list of 5,329 ABO_3_ perovskites can be found on figshare (Data Citation 1). All the calculations, along with all the 470,000 compounds used for the stability calculations are available for download or for direct consultation at www.oqmd.org. Kirklin *et al.*^8^ also contains detailed information about the calculation parameters.

### File format

The data is stored in a CSV spreadsheet. Each row contains a different composition and each column is a property of that composition (described in Table 2). A calculation that did not converge to a final solution is indicated by a hyphen (‘−’) in the table for that composition. This lack of convergence can happen for variety of reasons, for instance an initial geometry that is very far from the final relaxed geometry, a chemistry consisting of elements for which electronic self-consistency is difficult to achieve, etc. These kinds of computational issues are commonplace in high-throughput methods where *consistent settings* have to be used for the calculation of *all* compounds in a reasonable amount of time.

### Graphical representation of the data

The top part of Fig. 1 shows the number of stable perovskites as a function of the elements occurring on the A- and B-sites. Out of 73 elements, only boron does not appear in any stable perovskites. Lanthanides and alkaline earths are frequently on the A-site for stable perovskites whereas transition metals, specially the first row, are common on the B-site. These elemental preferences are consistent with what is experimentally reported in the literature^1,26^.

The bottom part of Fig. 1 shows the formation energy and band gap distribution for all the compounds calculated in this work. We see that most compounds have negative formation energies (only 276 are positive). The difference between negative formation energy and stability is apparent as the majority of materials have a negative formation energy but are not stable. As for the band gaps, the majority of them are 0 which is expected when using GGA-PBE (see band gap paragraph in the technical validation section).

## Technical Validation

### The open quantum materials database

The Open Quantum Materials Database uses density functional theory (DFT) to compute the total energies of every compound. DFT is widely used in solid states physics due to its accuracy and reproducibility^30–32^. In addition, previous studies have shown that formation energies calculated using DFT, when compared against those measured experimentally, have a similar accuracy as a comparison between experimental values from two different sources^8^.

### Lattice parameter

**Figure 3:**
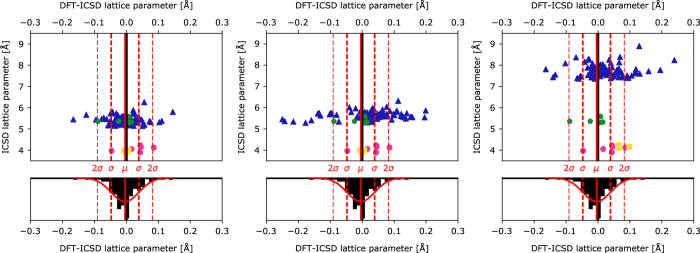
Comparison between DFT and ICSD lattice parameters for 113 compounds. (left) lattice parameter a, (center) lattice parameter b, and (right) lattice parameter c. In the top panels, the horizontal axes measure the difference between the computed and experimental lattice parameters while the vertical axes are the experimental lattice parameters. The lower plots correspond to a histogram of the difference in lattice parameters from DFT and experiment. The solid and dashed red lines indicate the average error, first and second standard deviations between DFT and experiment, respectively.

For all the compounds that are predicted to be stable and have an entry in the ICSD, we compared the lattice parameters of the DFT relaxed structure with the lattice parameter of the experimental structure (Fig. 3). The mean error (ME), mean absolute error (MAE), mean relative error (MRE) and mean absolute relative error (MARE) across all lattice parameter for the 113 compounds are 0.011 Å, 0.048 Å, 0.20 and 0.82%, respectively. The magnitude and overestimation of the lattice parameters are consistent with other lattice parameters studies in the literature for DFT-PBE^33^.

### Supercell size for oxygen vacancy formation energy

**Figure 4:**
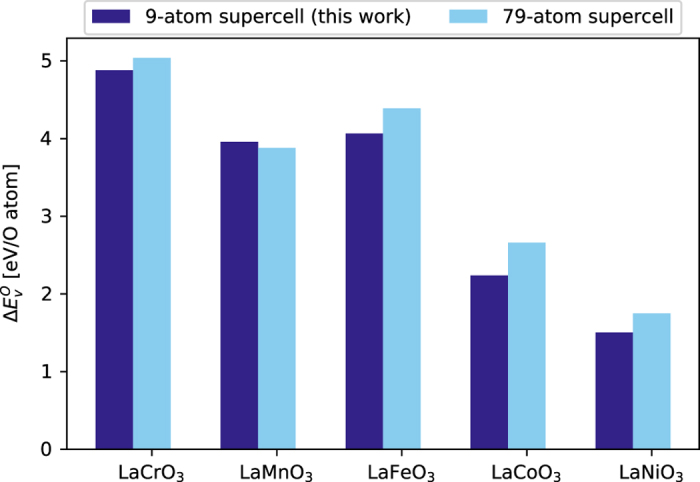
Comparison of oxygen vacancy formation energy between the present work (9-atom supercell) and larger supercells (79-atom). Data for the 79-atom supercell are taken from Deml *et al.*^34^

We compared our oxygen vacancy formation energies calculated using 9-atom supercells with oxygen vacancy formation energy calculated using larger supercell sizes (79-atom)^34^. We see good agreement between our high-throughput approach and data from the literature (see Fig. 4). In addition, Curnan and Kitchin^35^ have showed that oxygen vacancy formation energy trend is largely unaffected by the supercell size for LaBO_3_ and SrBO_3_ (B=Sc-Cu).

### Band gap

The band gaps were calculated with GGA-PBE, with U-values for some 3d-transition metals and actinides (see Table 1). GGA-PBE tends to underestimate the band gaps of semiconductors^36,37^ meaning that band gap values presented in this work have to be taken as lower bound and are useful to identify insulators. Different, much more expensive, calculations, such as hybrid functionals or quasiparticle calculations (G_0_W_0_, GW_0_ and GW), can be done to compute band gap values more accurately^36^.

### Magnetism

Several perovskites are experimentally observed to have complex magnetic structures, e.g., antiferromagnetic order^38^. However, in order to save computational times as the magnetic ordering of an unknown structure cannot be known *a priori*, only ferromagnetic configurations are calculated in the present study. Stevanović *et al.*^39^ showed that the error associated with different magnetic ordering is of the order of 0.01 to 0.02 eV per atom by calculating ternary compounds with up to ten different spin orderings. To take this potential error into consideration, compounds with a hull distance below 0.025 eV per atom are labelled as stable in this study.

### Comparison with experimentally observed perovskites

Of the 5,329 different compositions that were calculated, 395 are predicted to be thermodynamically stable by density functional theory. Out of those, 165 are reported in the literature. As a result, 230 *new* compounds are predicted to be DFT stable but not yet experimentally reported. This set of compounds represents a wide range of predictions amenable for materials synthesis.

**Figure 5:**
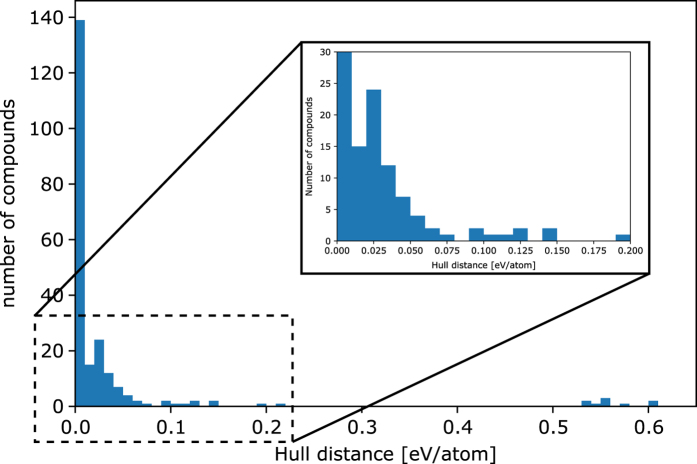
Histogram of the DFT stability of 223 ABO3 perovskite compounds reported in the literature. The inset shows the rapid decay of stable compounds as a function of stability.

The stability values of the 223 compounds that we found in 4 review papers^26–29^ are plotted in Fig. 5 (for clarity, all stable compounds are merged into the ‘0 hull distance bin’). The plot shows that a large number of these experimentally reported compounds are stable according to our DFT T=0 K calculations. However, the remainder of the phases are above the convex hull, and hence metastable (or unstable). The results of Fig. 5 shows the measure of metastability in terms of convex hull distance: there is rapid decay of the number of synthesized compounds as the convex hull distance increases, reaching almost 0 at a hull distance of 0.1 eV per atom. This 0.1 eV/atom metric for metastability is consistent with the results from another recent high-throughput study of metastabiity by Sun *et al.*^40^

Nine compounds reported in the literature are seen with a stability above 0.5 eV per atom. All these compounds contain rare earth elements, which are difficult to treat accurately with DFT because of the complexities associated with f-electron systems. In our high-throughput study, f-electrons are not included in the valence electrons of the pseudopotentials used, and therefore the DFT calculations of rare-earth-containing perovskites could have physical errors associated with the approximations made in the DFT calculations. For a more detailed discussion about f-electrons and frozen-core potentials, we refer the reader to Kirklin *et al.*^8^ Errors can also come from erroneous experimental characterization and/or classification.

## Usage Notes

We suggest using the data as it is in the spreadsheet. If one chooses to access the data from OQMD via qmpy, we note that the OQMD is a constantly-growing database. Indeed, as a result of compounds being constantly calculated and added to the database, the stability of the already-present compounds can change: adding new stable compounds may change the predicted stability of a perovskite.

## Additional Information

**How to cite this article:** Emery, A. A. & Wolverton, C. High-throughput DFT calculations of formation energy, stability and oxygen vacancy formation energy of ABO_3_ perovskites. *Sci. Data* 4:170153 doi: 10.1038/sdata.2017.153 (2017).

**Publisher’s note:** Springer Nature remains neutral with regard to jurisdictional claims in published maps and institutional affiliations.

## Supplementary Material



## Figures and Tables

**Table 1:** U-values used for the calculations of compounds containing the listed elements.

**Element**	**U-value [eV]**
V	3.1
Cr	3.5
Mn	3.8
Fe	4
Co	3.3
Ni	6.4
Cu	4
Th	4
Pa	4
U	4
Np	4.0
Pu	4.0

**Table 2:** Description of column keys for the CSV spreadsheet containing the dataset (Data Citation 1).

**Name**	**Type**	**Unit**	**Description**
Chemical formula	string	None	Chemical composition of the compound. The first and second elements correspond to the A- and B-site, respectively. The third element is always oxygen
A	string	None	Chemical element on the A-site
B	string	None	Chemical element on the B-site
In literature	boolean	None	Report of experimental synthesis of compound in the literature. True indicates that the compound is present in one of the four review papers.
Valence A	number or string	None	Valence of atom A as estimated by bond valence (BV) theory. If a compound is not balanced, it is denoted by ‘not balanced’. If the compound contains a least one element without a BV parameter, it is denoted by ‘element not in BV’
Valence B	number or string	None	Valence of atom B as estimated by bond valence (BV) theory. If a compound is not balanced, it is denoted by ‘not balanced’. If the compound contains a least one element without a BV parameter, it is denoted by ‘element not in BV’
Radius A	number	Å	Shannon ionic radius of atom A. When possible, the oxidation state and coordination number (12) of the A atom was used to estimate its radius.
Radius B	number	Å	Shannon ionic radius of atom B. When possible, the oxidation state and coordination number (6) of the B atom was used to estimate its radius.
Lowest distortion	string	None	Distortion with the lowest energy (among cubic, rhombohedral, tetragonal and orthorhombic corresponding to space group 221, 167, 99 and 62, respectively)
Formation energy	number	eV per atom	Formation energy as calculated by equation (1) of the distortion with the lowest energy
Stability	number	eV per atom	Stability (hull distance) as calculated by equation (2) of the distortion with the lowest energy. A compound is considered stable if it is within 0.025 eV per atom of the convex hull
Magnetic moment	number	*μ*_*B*_	Resulting magnetic moment of the relaxed structure. If the composition does not contain any magnetic element, the magnetic moment is set to a hyphen (‘−’).
Volume per atom	number	Å^3^ per atom	Volume per atom of the relaxed structure
Band gap	number	eV	PBE band gap obtained from the relaxed structure
a	number	Å	Lattice parameter a of the relaxed structure
b	number	Å	Lattice parameter b of the relaxed structure
c	number	Å	Lattice parameter c of the relaxed structure
alpha	number	°	*α* angle of the relaxed structure. *α*=90 for the cubic, tetragonal and orthorhombic distortion.
beta	number	°	*β* angle of the relaxed structure. *β*=90 for the cubic, tetragonal and orthorhombic distortion.
gamma	number	°	*γ* angle of the relaxed structure. *γ*=90 for the cubic, tetragonal and orthorhombic distortion.
Vacancy energy	number	eV per O atom	Oxygen vacancy formation energy as calculated by equation (3)

## References

[d1] Figshare Emery A. A. Wolverton C. 2017 http://dx.doi.org/10.6084/m9.figshare.5334142

